# Supplementary material for: Lactulose Modulates the Structure of Gut Microbiota and Alleviates Colitis-Associated Tumorigenesis
Source: Nutrients. 2022 Feb 3;14(3):649. doi: 10.3390/nu14030649 (PMC8840163; doi:10.3390/nu14030649)
Supplement: Supplementary file 1 [file nutrients-14-00649-s001.zip › nutrients-1570025-supplementary.pdf]

A

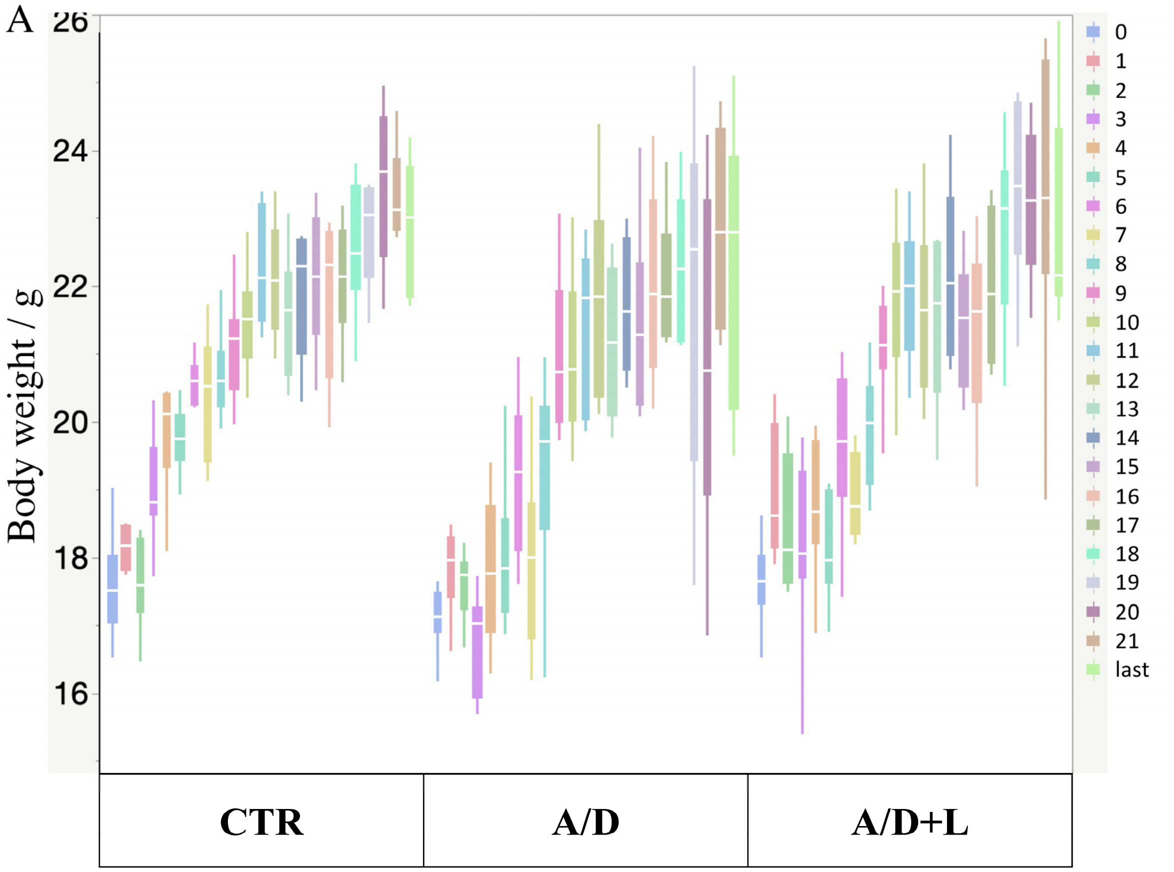

B

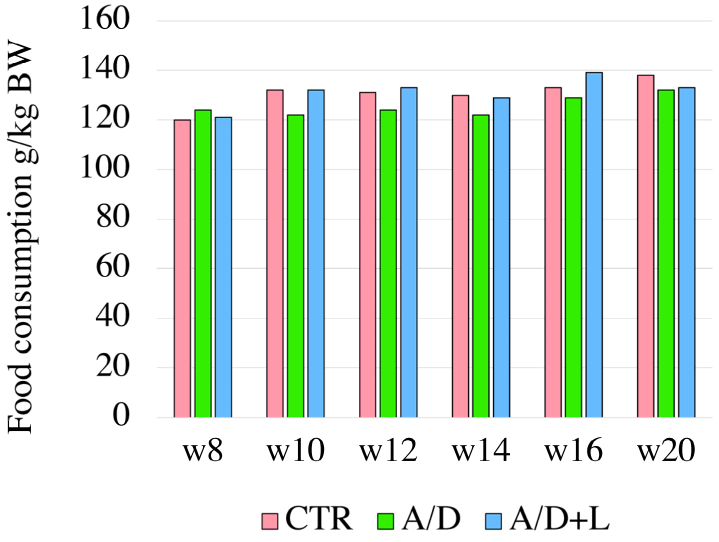

Supplementary Figure 1

Figure S1. The body weight and food consumption. in the backsection. (**A**) The body weight changes of 3 groups (n = 8); (**B**) Food consumption of 3 groups from 8 weeks to 20 weeks (n = 8).
